# Preparation of hafnium nitride-coated titanium implants by magnetron sputtering technology and evaluation of their antibacterial properties and biocompatibility

**DOI:** 10.1515/biol-2025-1132

**Published:** 2025-07-24

**Authors:** Jun Xue, Yi Jiang, Jinyu Zhu, Sihui Chen

**Affiliations:** Department of Orthopaedics, Jiaxing University Affiliated Hospital, The First Hospital of Jiaxing, South Central Avenue No. 1882, Jiaxing, 314000, Zhejiang, China

**Keywords:** titanium implants, hafnium nitride coatings, *Staphylococcus aureus*, antimicrobial properties, biocompatibility

## Abstract

Hafnium nitride (HfN) coatings with different thicknesses and various composition ratios were successfully fabricated on the surface of titanium alloys by magnetron sputtering, and their antibacterial properties and biocompatibility were evaluated. The structures of the coatings were characterized by X-ray diffraction (XRD; DX-2700BH diffractometer) in symmetric *θ*–2*θ* scanning mode. The surface morphologies of the coatings were characterized by scanning electron microscopy (SEM; Nova NanoSEM450). The surface element distribution of the film was characterized by the energy-dispersive spectrometer (EDAX ELECT PLUS). The antibacterial properties of different materials against *Staphylococcus aureus* (ATCC 25923) were evaluated by plate colony counting, crystal violet staining, SEM, and SYTO-9/PI live/dead bacterial staining. The proliferation of bone marrow mesenchymal stem cells was detected by CCK-8 assays, and the biocompatibilities of different materials were evaluated by calcein-acetoxymethyl/propidium iodide (PI) live/dead cell staining. The surface morphology and element analyses revealed that the HfN coatings and titanium substrates did not contain other substances, and their surfaces were relatively uniform. After 24 h incubated with *Staphylococcus aureus*, sample 2 (50 nm thick, N_2_ flow rate of 2.5 sccm) displayed the best antibacterial performance. CCK-8 cell proliferation and calcein-AM/PI live/dead cell staining assays indicated that sample 2 had the best biocompatibility. The modified titanium implants had good biocompatibility and antibacterial properties. The results presented here can potentially guide and inspire additional ideas to alter the surfaces of titanium implants.

## Graphical abstract

**Figure:**
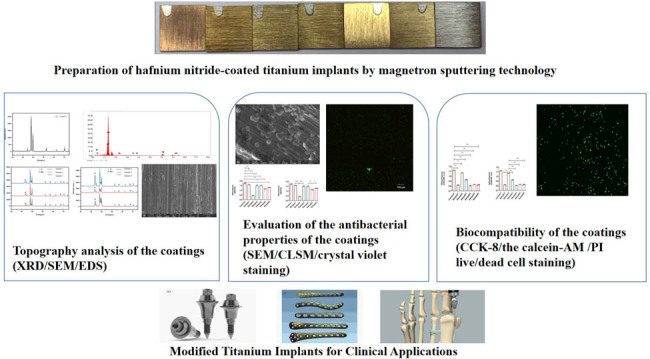

## 1 Introduction

Orthopedic implants have been widely used for fracture trauma fixation, spinal fusion internal fixation, and artificial joint replacement because they can relieve the pain of patients; however, they also carry the risk of infection [1]. An important mechanism of orthopedic implant infection is the formation of a bacterial biofilm, which greatly extends the treatment cycle and increases the difficulty of treatment [2,3]. Therefore, preventing implant infection is particularly important, which has made anti-infection orthopedic implants a research hotspot.

Due to its superior biocompatibility, good machinability, strong osseointegration ability, density, and elasticity that match human bone, titanium (Ti) has attracted much attention in biomedicine, such as dentistry, orthopedics, and orthopedic implants [4,5]. However, a 2–5 nm thick surface oxide layer, mainly composed of TiO_2_, readily forms on the surface of pure titanium implants, which can affect the osteointegration and biocompatibility of the implant [6,7]. In addition, Ti and its alloys do not have antibacterial properties. After implantation into the human body, bacteria can easily accumulate and eventually form a biofilm, which resists antibacterial treatment and hinders osseointegration [8,9]. Ti and its alloys are the most commonly used materials for permanent implants that contact bone, so preventing infections on the surface of Ti implants is a major challenge for orthopedic surgeons.

Previous studies have shown that the surface parameters of titanium-based alloys, including the roughness, morphology, and elemental composition, affect the infectivity, cell proliferation, cell adhesion, and gene expression of the tissues surrounding Ti implants, thereby affecting the bone healing process [10,11]. The currently used antibacterial surface modification methods can be divided into two types: surface topography construction and the formation of an antibacterial coating on the surface [12,13,14,15,16].

Magnetron sputtering is a commonly used thin film preparation technique that utilizes the principles of ion bombardment and sputtering by applying high-frequency electric and static magnetic fields in a vacuum environment [17]. Compared with traditional sputtering technology, magnetron sputtering has a higher ionization efficiency and a more stable plasma environment, so it can achieve a more efficient and uniform coating process. We use this technology to form an antibacterial coating on the surface of titanium substrates.

The periodic table element, hafnium, falls into the same period, similar to standard titanium. Hafnium has been researched as an alloy combination or coating in various *in vitro* scenarios [18,19,20]. Hafnium is also similar to titanium in its behavior with osseous tissues [19,20]. This hafnium metal coating of commercially available titanium implants has proven to show some superior properties to titanium with *in vitro* studies and soft tissue [21]. Hafnium nitride (HfN) is a typical covalently bound material with free electrons, high corrosion resistance, and good mechanical properties. It can maintain its good surface characteristics after 10,000 friction and wear tests [22,23]. According to our previous research, HfN has a small corrosion current density (5.09 × 10^−4^ A/cm^2^, 4.64 × 10^−4^ A/cm^2^) in 0.5 mol/L H_2_SO_4_ and 3.5 wt% NaCl solution, indicating that HfN membrane has excellent acid and salt corrosion resistance [24]. The surface roughness of HfN film was increased from 0.92 to 2.93 nm. The static water contact angle only increased from 98.1° to 106.6°, indicating a hydrophobic state [24], and it can reduce the risk of osteolysis. HfN coatings have potential as an osteoinductive materials, and its advantages are mainly reflected in the excellent biocompatibility and chemical stability, surface modification to enhance osseointegration, mechanical properties adapted to bone tissue, immune regulation, and so on [25,26]. HfN has shown significant antibacterial potential by multiple mechanisms such as metal ion release, oxide layer catalysis, and nanostructure effect [21,27]. However, further research is needed to optimize its clinical application feasibility.

This study is the first attempt to replace traditional microfabrication technologies, such as ion etching, with magnetron sputtering, construct a novel HfN nanostructured antibacterial coating on the surface of an orthopedic Ti implant, and explore its antibacterial properties and biocompatibility.

## 2 Experimental details

### 2.1 Materials and preparation

A pure Ti (TA1 grade) sheet with a diameter of 10 mm × 10 mm × 1 mm was used as the matrix; continuously washed with acetone, ethanol, and deionized water under high-frequency ultrasonic; and then dried for later use. In the atmosphere of N_2_ and Ar, HfN films of different thicknesses and different composition ratios were deposited on the titanium substrate by sputtering a pure hafnium target (purity 99.99%). The thicknesses of coatings were 50 and 200 nm. The compositional ratio of HfN was achieved by controlling the flow ratio of Ar and N_2_; the thickness was controlled by the sputtering time in Table 1.

**Table 1:** Process parameters by magnetron sputtering technology

Number	Parameters
Control 1	Ti
Sample 1	HfN, 150 W, Ar 80 sccm, N_2_ 3.0 sccm, 50 nm, 12 min 30 s
Sample 2	HfN, 150 W, Ar 80 sccm, N_2_ 2.5 sccm, 50 nm, 13 min 3 s
Sample 3	HfN, 150 W, Ar 80 sccm, N_2_ 2.0 sccm, 50 nm, 13 min 38 s
Sample 4	HfN, 150 W, Ar 80 sccm, N_2_ 3.0 sccm, 200 nm, 50 min
Sample 5	HfN, 150 W, Ar 80 sccm, N_2_ 2.5 sccm, 200 nm, 52 min 10 s
Sample 6	HfN, 150 W, Ar 80 sccm, N_2_ 2.0 sccm, 200 nm, 54 min 33 s

The radio frequency power was 150 W; the Ar traffic was always 80 sccm; and the N_2_ traffic was 2, 2.5, and 3 sccm, respectively. In this way, the purpose of changing the coating composition ratio was achieved. The background vacuum was 4 × 10^−4^ Pa, the working pressure was 1.1 Pa, the stage speed was 5 rpm, and the bias voltage was −80 V. The samples were divided into seven groups according to the different coating thickness and composition ratio, as shown in Table 1. The prepared sheets were wiped with 75% alcohol on a sterile bench and sterilized by ultraviolet irradiation for 15 min.

The crystal structure of the coatings was characterized by an X-ray diffractometer (XRD, DX-2700BH diffractometer, Dandong, China) in symmetric *θ*–2*θ* scanning mode. The surface element distribution of the film was characterized by the energy-dispersive spectrometer (EDS, EDAX ELECT PLUS,USA).

### 2.2 Biofilm formation on implants

This study used *Staphylococcus aureus* (*S. aureus*) ATCC 25923, provided by the Department of Clinical Laboratory, Jiaxing First Hospital. The strain was grown on tryptic soy agar (TSA; Fushenbio, Shanghai, China) at 37°C, 5% CO_2_. Then, representative colonies were picked and suspended in tryptic soy broth (TSB; Fushenbio, Shanghai, China), growing at 37°C overnight with agitation (200 rpm). Bacteria were harvested and resuspended in TSB, adjusted to a turbidity equivalent to that of a 1 McFarland standard, and diluted 1:300, achieving the final cell concentration of approximately 1 × 10^6^ colony forming units (CFU)/mL.

The sterile sheets were put in the 24-well clear bottom microtiter plates (Corning Inc, Corning, NY). Subsequently, 1 mL of bacteria suspension was added to each well and incubated for 24 h at 37°C, 5% CO_2_.

### 2.3 Colony plate counting

Each sample was rinsed with sterile phosphate buffered saline (PBS) after being incubated for 24 h. After being cleaned, the sample was put in a 15 mL tube with 10 mL of PBS. About 10 mL of PBS was subjected to a 35 kHz sonication frequency for 15 min to remove the biofilm. There was an additional 10 s vortex period added in between each of the three sonication cycles. Ten serial dilutions were prepared and plated onto TSA, which was subsequently incubated at 37°C with 5% CO_2_ for a full day. Next, the number of colonies observed on the TSA was summed, and the CFU was calculated based on the dilution.

### 2.4 Crystal violet method for biofilm detection

After incubation with the bacterial inoculum for 24 h, the solution was aspirated, and 1 mL of 0.1% crystal violet staining solution (Beyotime, Shanghai, China) was added to each well. The plate was then incubated at room temperature for 15 min to stain the biofilm on the material surface. Following stain removal, the wells were rinsed three times with PBS and air-dried. Subsequently, 1 mL of 95% ethanol was added to each well and incubated for 15 min to dissolve the biofilm. A microplate reader (ELX800, Bio-Tek, USA) was used to measure absorbance at a wavelength of *λ* = 570 nm. To obtain stable and accurate data, six replicates were set for each data to take the average value, and three tests were performed.

### 2.5 Confocal laser scanning microscopy (CLSM)

After 24 h incubation of the material and bacterial suspension, the bacterial solution was aspirated and washed twice in sterile PBS. SYTO-9/PI live/dead bacteria fluorescent stain (Fushen Biotech, Shanghai, China) was used to fluoresce the aforementioned samples, according to the instructions of the staining kit, stained at room temperature for 15 min, pay attention to protect from light. The stain was aspirated and rinsed with sterile PBS buffer, and the adhesion of live dead bacteria on the surface of the material was observed using a laser confocal microscope (CLSM, LSM800, ZEISS, Germany).

### 2.6 Scanning electron microscopy (SEM)

After 24 h of incubation, the samples were washed three times with PBS, fixed overnight with 1 mL of 2.5% glutaraldehyde solution, washed three times with PBS, and dehydrated with 30, 50, 70, 80, 90, and 100% ethanol solution for 15 min. The samples were placed in a 1:1 solution of ethanol and tert-butanol for 15 min and placed in a freeze dryer for freeze drying. SEM (Nova NanoSEM450, FEI, USA) was used to detect the adhesion of *S. aureus* on the surface of the sample.

### 2.7 Isolation and culture of bone mesenchymal stem cells (BMSCs)

BMSCs were obtained from the femurs and tibias of a 6-week-old female *Sprague-Dawley*. All animal experiments and protocols were approved by the Laboratory Animal Ethics Committee of Jiaxing First Hospital (permit number: JXYY2024-004). The culture medium was used to flush the bone marrow cavities of isolated femurs and tibias until the diaphysis turned completely white. Then, the resulting cell suspension was centrifuged at 1,200 rpm for 5 min. The cells were then resuspended with erythrocyte lysis buffer and left to settle for 1 min, and the centrifugation process was repeated at 1,200 rpm for 5 min. Finally, the cells were resuspended in Dulbecco’s modified Eagle medium (DMEM; Gibco, USA), which contained 10% fetal bovine serum (FBS; Gibco, USA) and 1% penicillin and streptomycin (PS; Gibco, USA).

After three passages, BMSCs can be obtained for experimentation. The sterilized samples were placed into 24-well clear bottom microtiter plates with 2 × 10^5^ digested BMSCs per well, and BMSCs were grown in 1 mL DMEM and seeded on the surface of the samples for 48 h.


**Ethical approval:** The research related to animal use has been complied with all the relevant national regulations and institutional policies for the care and use of animals, and has been approved by the Laboratory Animal Ethics Committee of Jiaxing First Hospital (permit number: JXYY2024-004).

### 2.8 Cell counting kit-8 assay

After BMSCs were incubated on sterile material for 48 h, the Cell counting kit-8 assay (CCK8; MCE, China) was used to determine cell viability. Medium containing 10% CCK-8 solution was added to each well at the indicated time. BMSCs were placed in the dark and incubated for 2 h. Then, absorbance was measured by the absorbance microplate reader (Bio-Tek, Winooski, USA) at a wavelength of 450 nm.

### 2.9 Calcein-acetoxymethyl/propidium iodide (PI) live/dead cell staining

Sterile samples were prepared by wiping the samples by 75% ethanol and then by UV irradiation for 30 min. After 48 h of culture on sterile substrates, adherent BMSCs were harvested by trypsinization (0.25% trypsin–EDTA) and adjusted to a final concentration of 1 × 10^5^ cells/mL in complete medium. The Calcein-AM/PI Live/Dead Cell Staining Kit (Solarbio, China) was used to stain the cell suspension according to the instructions. About 2 μL Calcein-AM was added into 1 mL of cell suspension and incubated at 37°C for 25 min in the dark; 2 μL of PI was added for staining at room temperature for 5 min in the dark. Fluorescence microscopy detected live cells (yellow-green fluorescence) as well as dead cells (red fluorescence) at 490 ± 10 nm excitation filters.

### 2.10 Statistical analysis

GraphPad Prism 5 software (GraphPad Prism Software, Inc.; San Diego, CA, USA) was used for statistical analysis. A two-tailed, unpaired Student’s *t*-test with equal variance was used. A *p*-value <0.05 was considered to indicate statistical significance.

## 3 Results

### 3.1 Topography analysis of the coatings

**Figure 1:**
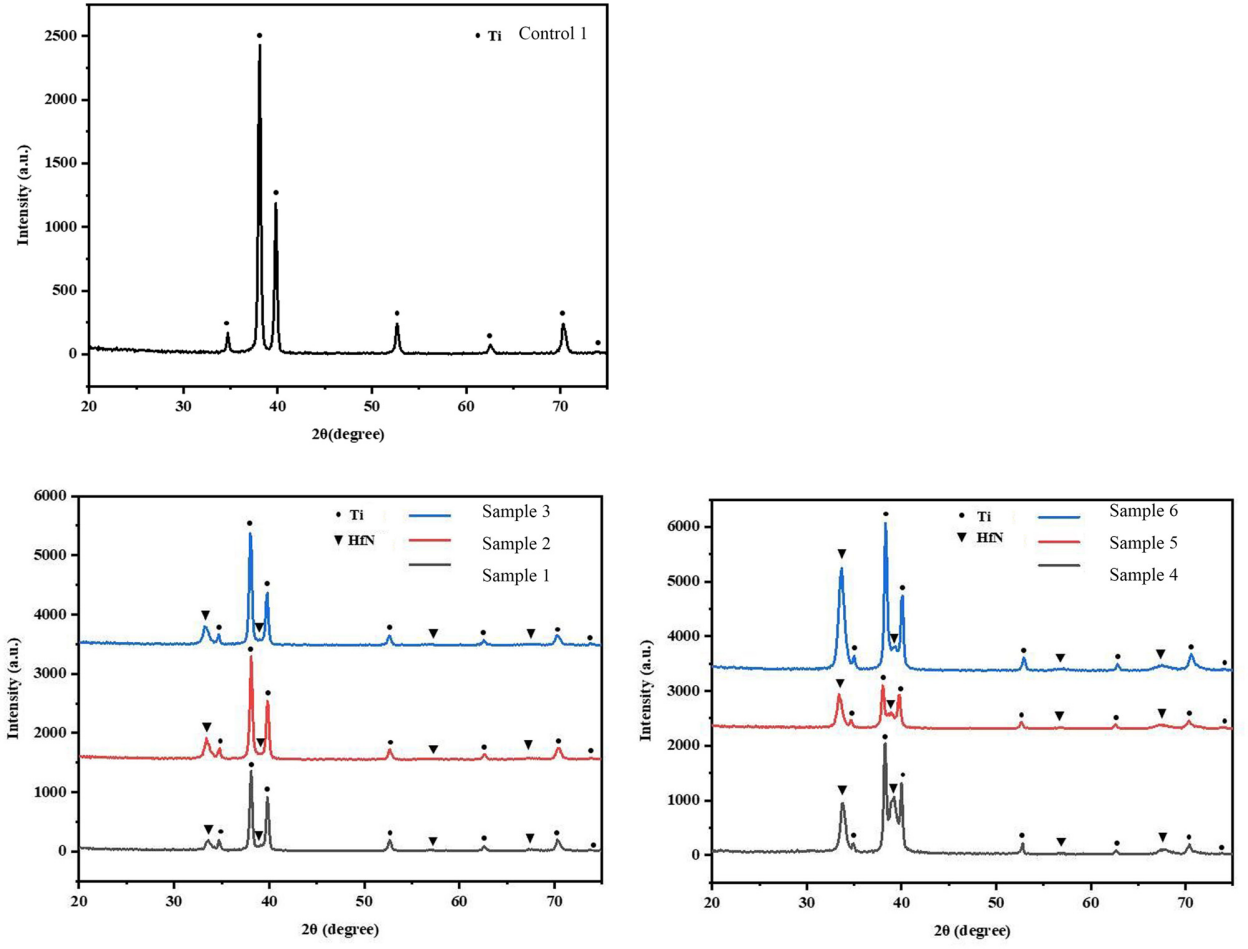
The crystal structures of the coatings were characterized by XRD in symmetric *θ*–2*θ* scanning mode.

**Figure 2:**
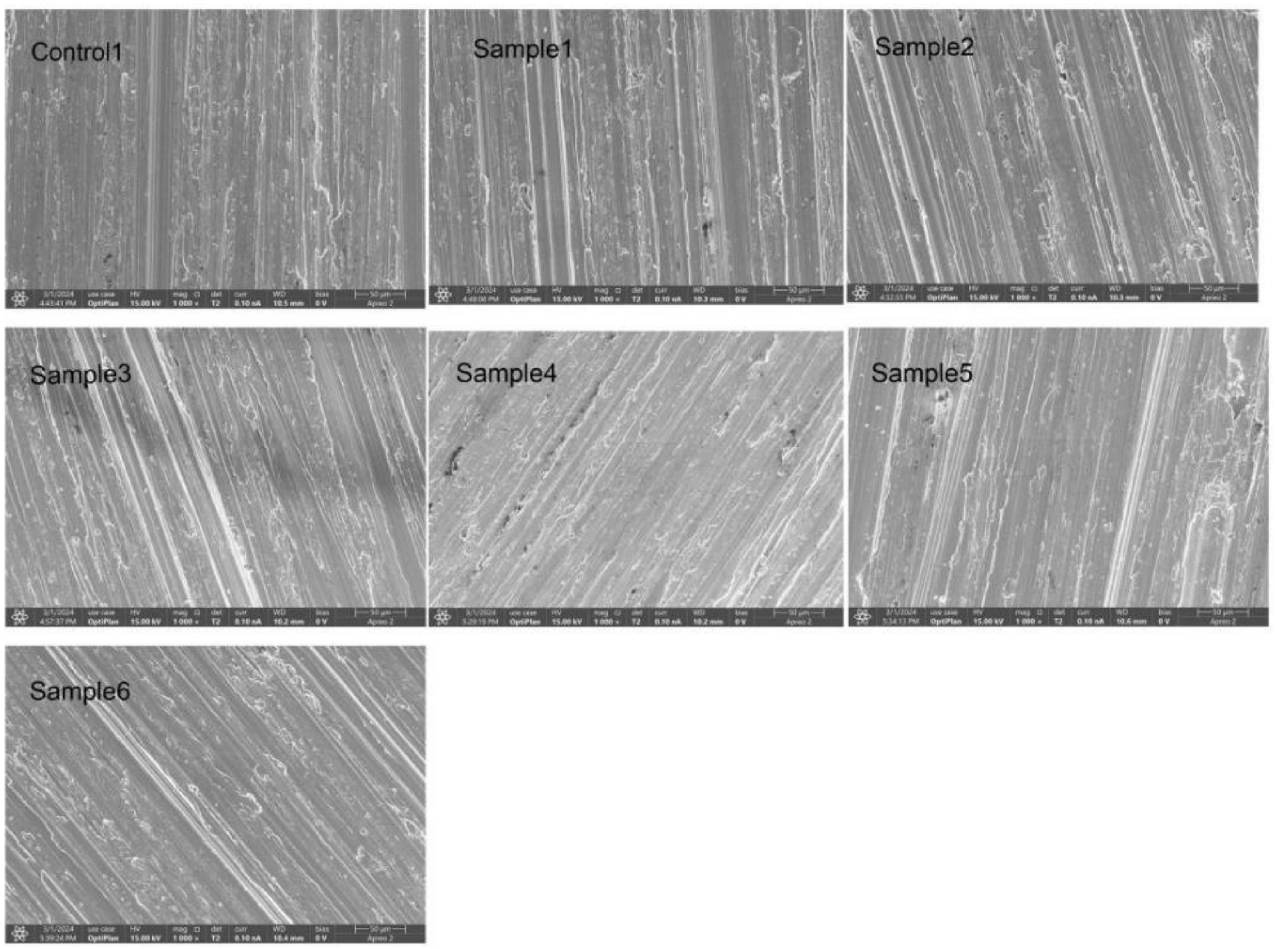
Surface topography of the material coating observed by SEM; scale bar = 50 μm.

XRD analysis provides crucial indirect evidence for evaluating coating uniformity. As demonstrated in Figure 1, the characteristic peaks of the Ti substrate show significant attenuation, indicating complete coverage by the HfN coating (X-ray penetration depth ∼1–5 μm). This observation is further corroborated by SEM characterization (Figure 2), which reveals a continuous coating morphology without exposed substrate areas. Our findings align with the methodology employed by Xu et al. [28]. In their study of nano-silver coatings on titanium substrates, combined XRD and SEM analysis was used to verify complete surface coverage. Based on this comprehensive characterization approach, we conclude that the HfN coating forms a uniform and continuous layer over the entire Ti substrate surface.

**Figure 3:**
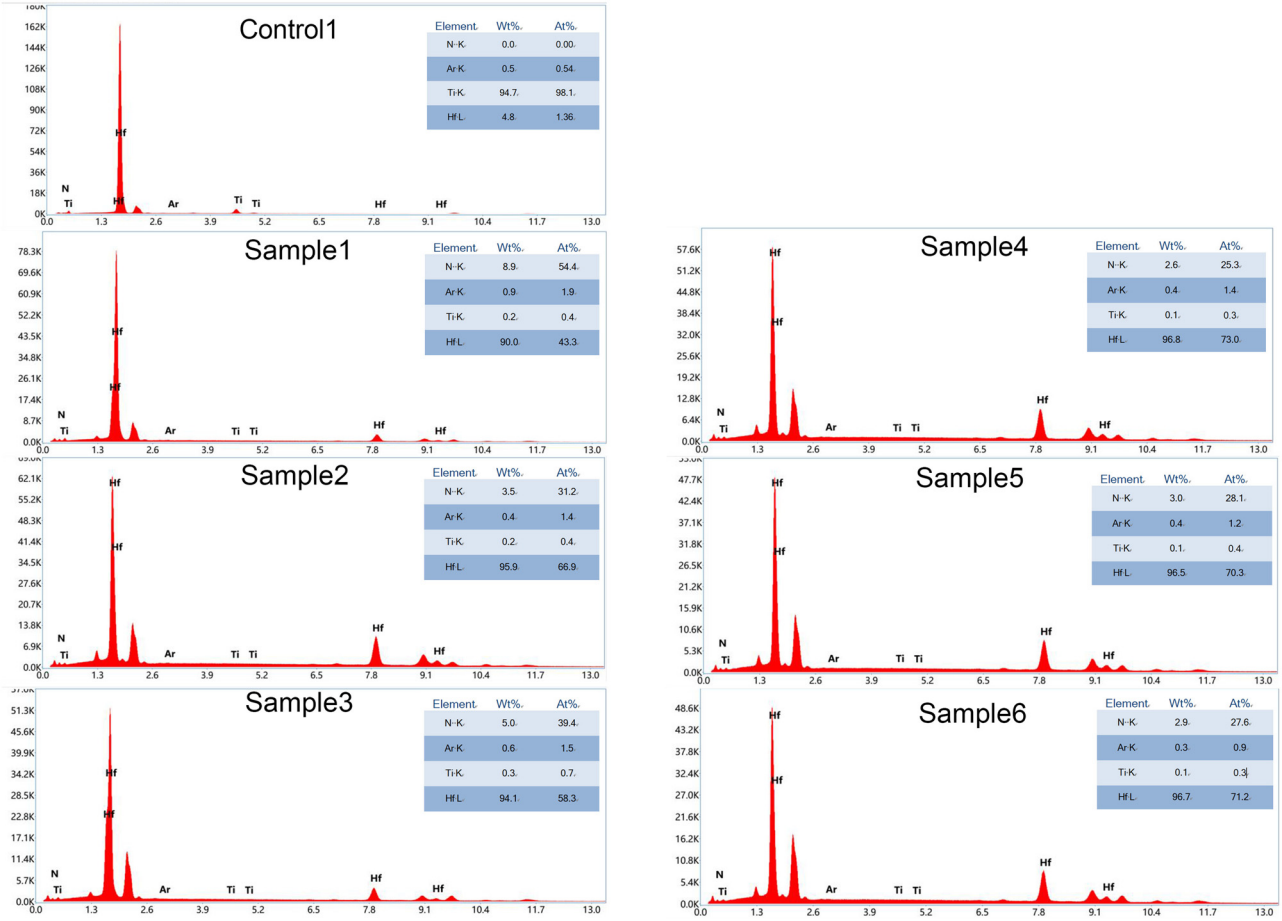
Surface element distribution of the film was characterized by EDS after magnetron sputtering with different parameters. wt%: weight percentage; at%: atomic percentage.

Figure 3 is obtained by EDS elemental mapping. EDS analyzed the weight percentage and atomic percentage of key elements on the coating surface. From Figure 6, it could be found that Ti and HfN were detected in all the samples, proved that 50 and 200 nm thicknesses of coatings magnetron sputtering could successfully attach different compositional ratio of HfN to the surfaces of the samples.

### 3.2 Evaluation of the antibacterial properties of the coatings

**Figure 4:**
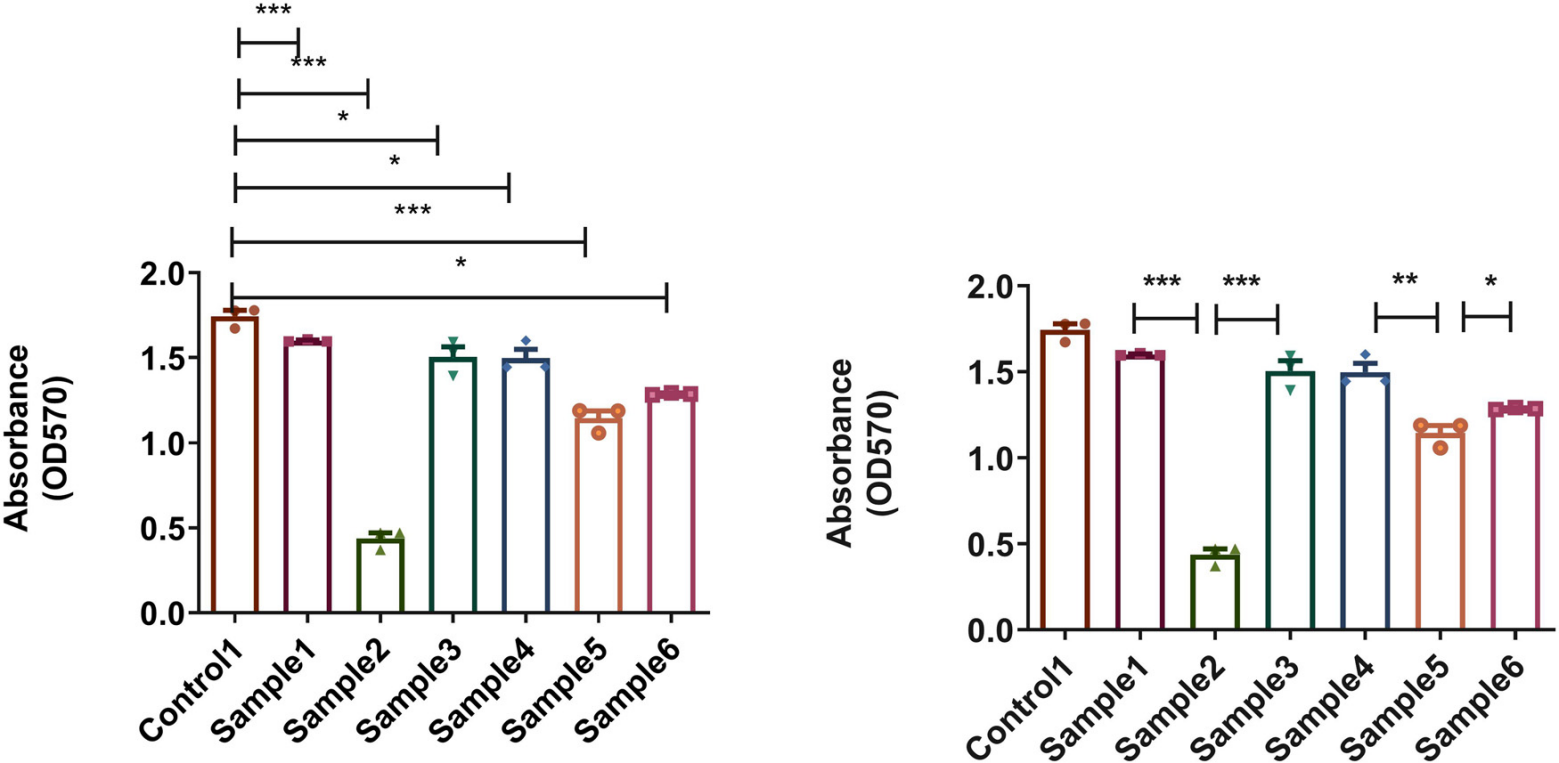
Quantitative results of crystal violet staining of the surfaces of biofilms on the implant material (*p < 0.05; **p < 0.01, ***p < 0.001).

In Figure 4, crystal violet staining showed that biofilm formation on the surfaces of sample 2 (with a thickness of 50 nm and an N_2_ flow rate of 2.5 sccm) and sample 5 (with a thickness of 200 nm and an N_2_ flow rate of 2.5 sccm) was significantly inhibited. Among them, the surface of sample 2 had the least amount of biofilm. Notably, the antibacterial properties of sample 2 was better than those of samples 1 and 3, and the antibacterial properties of sample 5 was better than those of samples 4 and 6, suggesting that the antibacterial properties of N_2_ flow at 2.5 sccm may be better than those of N_2_ at 2 sccm or 3 sccm.

**Figure 5:**
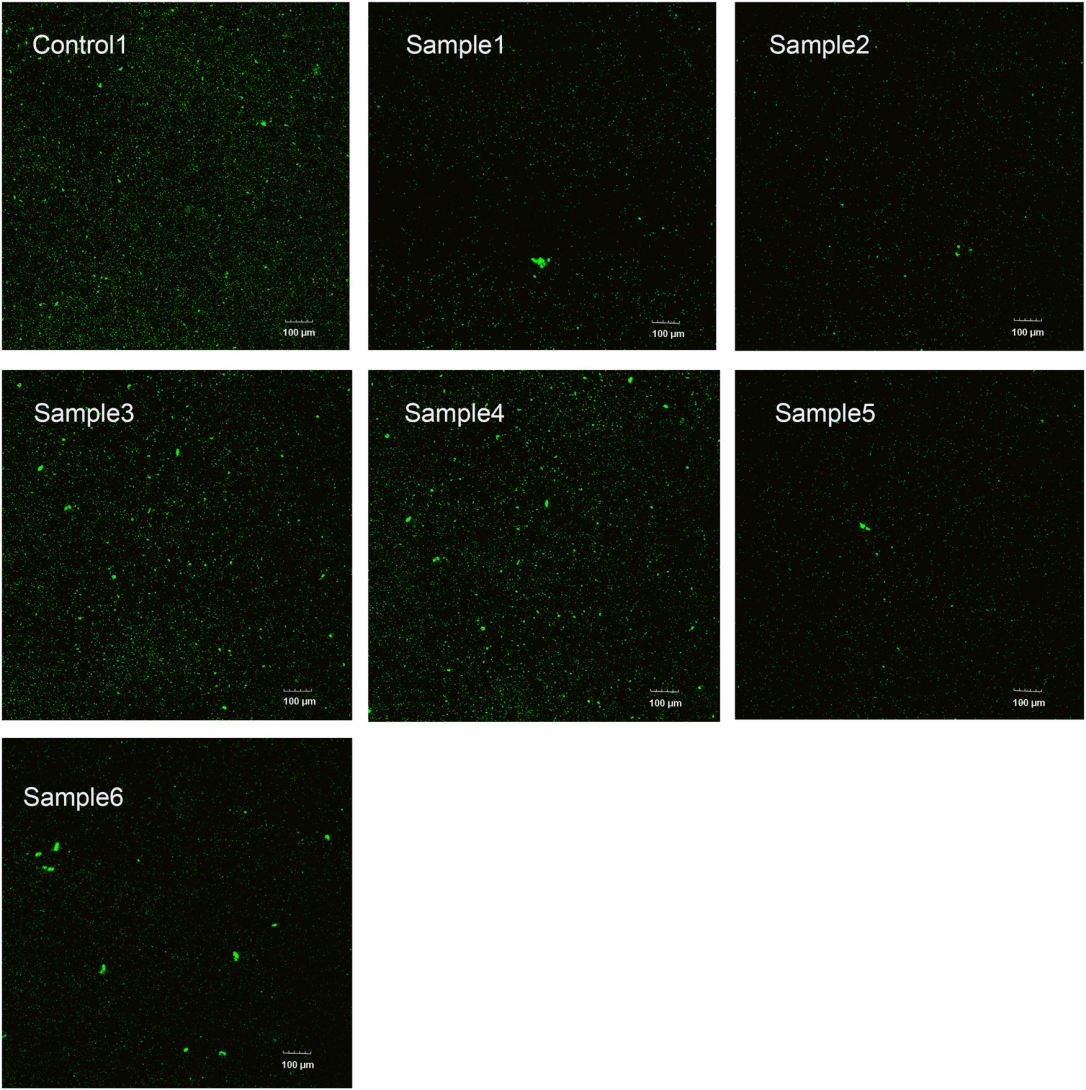
Control 1 and samples 1–6 were stained with SYTO-9/PI. SYTO-9-stained live bacteria to produce green fluorescence, while PI stained dead bacteria to produce red fluorescence; scale bar = 100 μm.

In Figure 5, SYTO-9/PI live/dead bacterial fluorescence staining indicated that the surface of sample 2 had less green fluorescence than those of control 1 and other samples. It is illustrated that the surviving *S. aureus* in sample 2 was less than other samples.

**Figure 6:**
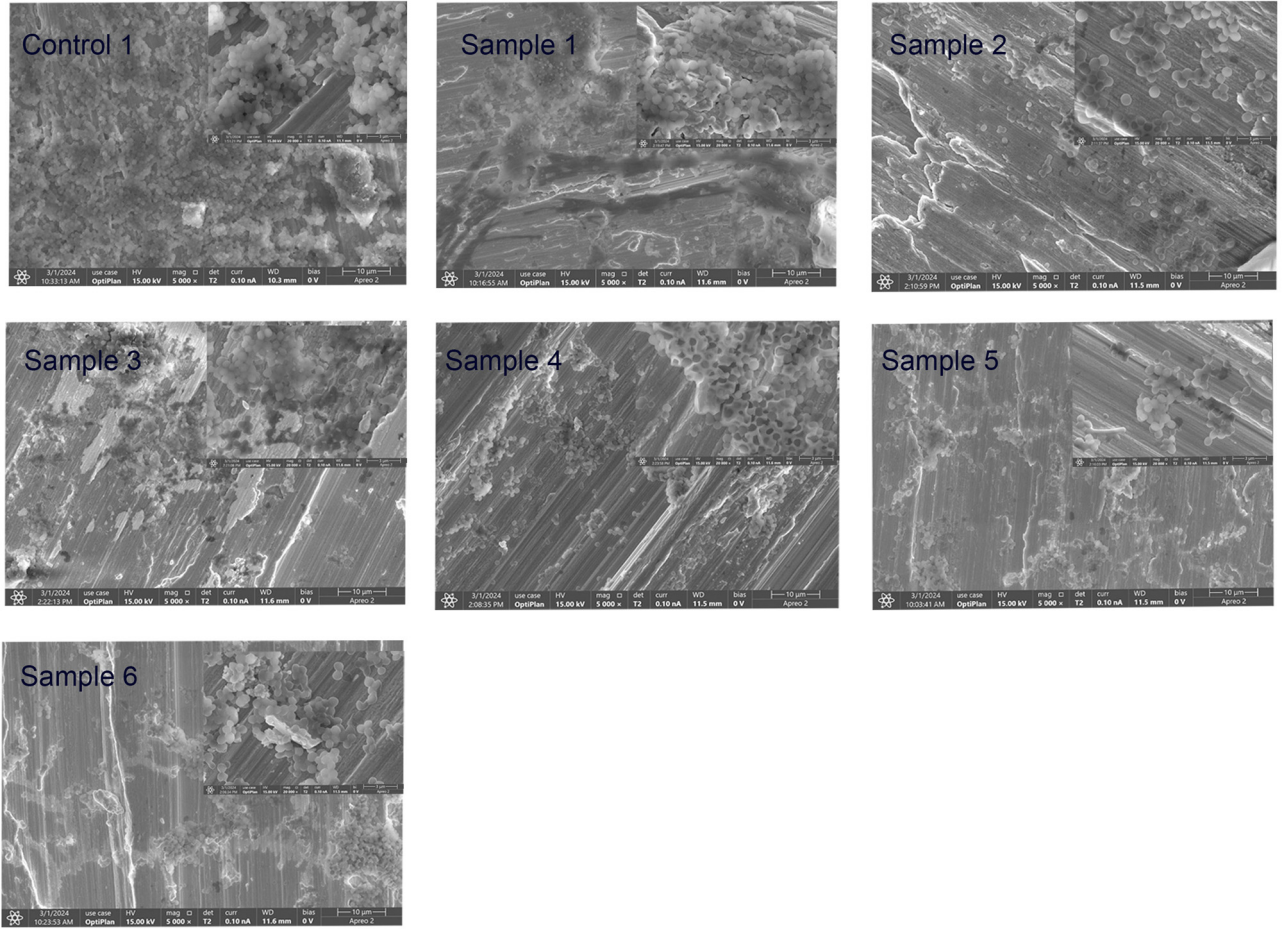
SEM was used to detect bacterial adhesion to the surface of the coating: a large scale bar = 10 μm and a small scale bar = 3 μm.

SEM was used to compare bacterial adhesion to the coating surfaces in Figure 6. Notably, most *S. aureus* adhered to the surface of control 1, while the least bacteria were found on samples 2 and 5. In Figure 5, the bacteria adhered to the surface of sample 2 were relatively scattered and not stacked, while those on the surface of sample 5 were stacked and accumulated to form aggregates. Moreover, the SEM results showed that the morphology of *S. aureus* remained unchanged.

### 3.3 Biocompatibility of the coatings

**Figure 7:**
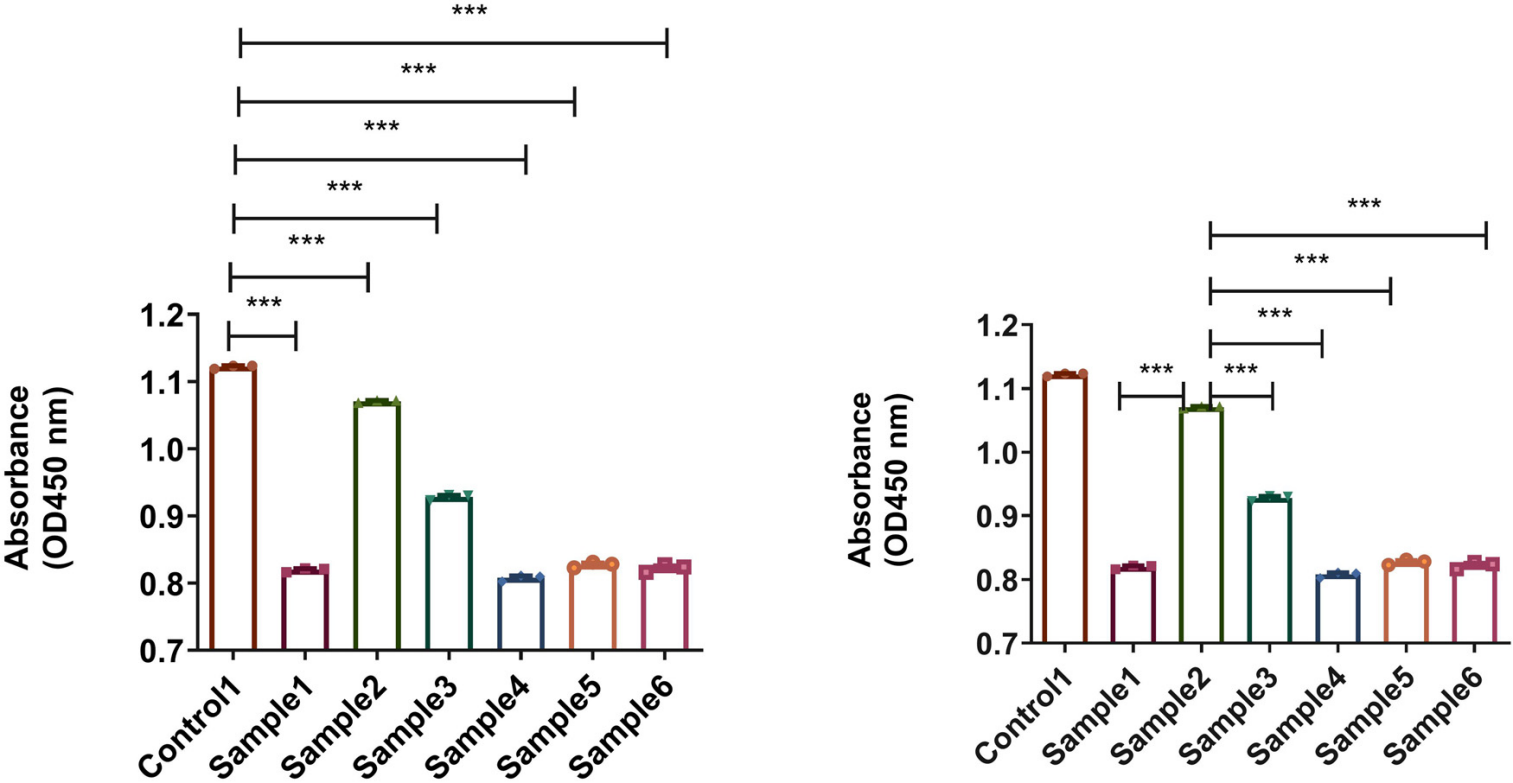
Viability of BMSCs cultured on control 1 and samples 1–6 was determined by CCK-8 assays (***p < 0.001).

After 2 days of coculture with the implant material, the CCK-8 method was used to analyze the proliferation of BMSCs, as shown in Figure 7. Compared to control 1, there was no decrease in cell viability on the coated surface of sample 2, while cell viability on the surface of the other samples decreased. In addition, the cell activity on the surface of sample 2 was higher than that of other samples.

**Figure 8:**
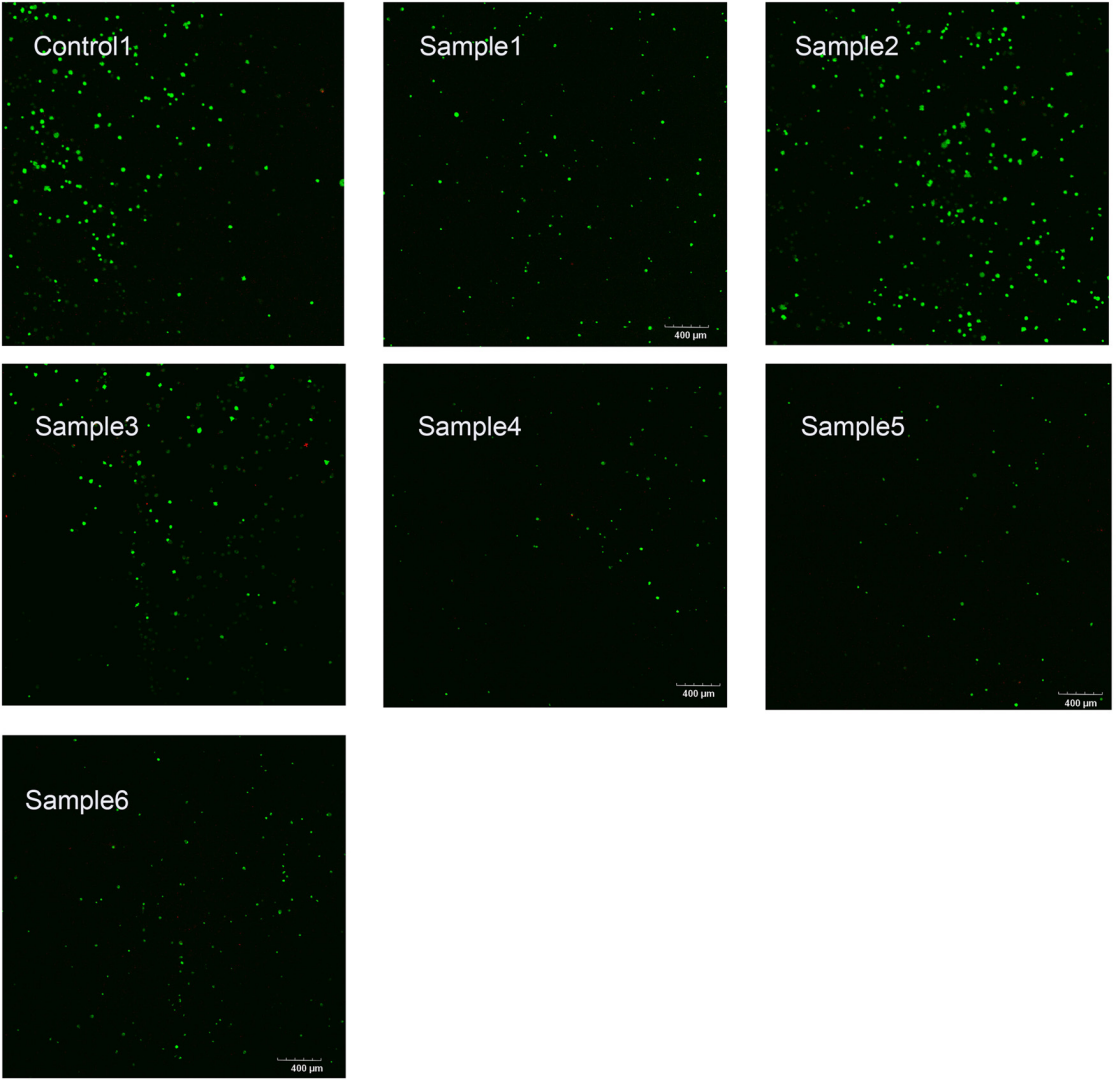
Calcein-AM/PI live/dead cell staining results. Calcein-AM fluoresces green in live cells, and PI fluoresces red in dead cells; scale bar = 400 μm.

Moreover, the calcein-AM/PI live/dead cell staining results showed that sample 2 had the highest number of viable cells compared to control 1 and other samples in Figure 8. It was consistent with the CCK-8 assay and indicated that sample 2 had the best biocompatibility.

## 4 Discussion

One of the most serious complications after the implantation of orthopedic materials is the development of an infection, which can cause clinical problems and patient suffering [28]. Thus, developing biomaterials with anti-infective properties is highly important for the prevention and treatment of related infections [29,30].

Graphene has been studied for its antimicrobial and coating applications [31], and the antibacterial, osteogenic, and angiogenic properties of the novel pH-responsive CaO_2_@ZIF-67-HA-ADH coating on titanium implants have also been investigated [32]. Ti and its alloys have been widely used in industry and the biomedical field, particularly for bone fusion, bone fixation, and joint replacement surgery [6]. Many researchers have modified the surface of titanium with antibiotics, antibacterial peptides, inorganic antibacterial metal elements, and antibacterial polymers to increase its antibacterial properties [33–35].

This is the first study applying magnetron sputtering technology to form an HfN coating on the surface of Ti. Grossman developed a classic method to quantify *S. aureus* biofilm formation by crystal violet staining and confocal microscopy, in an approach that has been standardized [36]. We used the same methodology to test the antibacterial properties of our coatings.

The SEM analysis showed that the HfN coating and Ti substrate formed relatively uniform nanoscale filaments, combined the XRD analysis shows the uniformity of the coating. The SEM analysis of bacteria adherence to the surface of the material revealed that the morphology of *S. aureus* was unchanged, indicating that the coating may have produced an antibacterial effect by inhibiting bacterial growth rather than killing the bacteria by changing their state, similarly inhibiting the growth of bacteria on material surfaces [37]. It is also possible that the dead *S. aureus* is not adherent therefore barely visible on the surface of the samples.

CCK-8 and live/dead cell staining assays have been used in many studies to evaluate biocompatibility [38]. Additionally, BMSCs are widely used in various orthopedic studies [39,40]. We also used these methods here to demonstrate that sample 2 did not exhibit significant toxicity to BMSCs. According to the calcein-AM/PI live/dead cell staining results, there were obvious live cells (green fluorescence), but not obvious dead cells (red fluorescence), indicating that the dead BMSCs may have been washed away without adhering.

Our study revealed that HfN coatings with a thickness of 50 nm and an N_2_ flow rate of 2.5 sccm had the greatest antibacterial effect on *S. aureus* compared to the other coatings studied in the present manuscript. In addition, SEM was used to detect bacterial adhesion to the surface of the materials, and the results were consistent. Notably, the antibacterial properties of sample 5 are second only to those of sample 2, indicating that the antibacterial performance of the N_2_ flow of 2.5 sccm may be better than that of the N_2_ flow of 2 sccm or 3 sccm. The EDS results showed that the composition of HfN element on the coating surface also changed accordingly. At 50 nm thickness, the Hf wt% of 2.5 sccm N_2_ flow was 95.9 and the Hf at% of 2.5 sccm N_2_ flow was 66.9, which was higher than 2 sccm or 3 sccm N_2_ flow; the N wt% of 2.5 sccm N_2_ flow was 3.5, and the N at% of 2.5 sccm N_2_ flow was 31.2, which was lower than 2 sccm or 3 sccm N_2_ flow. But at 200 nm thickness, the Hf wt% of 2.5 sccm N_2_ was 96.5 and the Hf at% was 70.3, which was lower than 2 sccm or 3 sccm N_2_ flow; the N wt% of 2.5 sccm N_2_ was 3.0 and the N at% was 28.1, which was higher than 2 sccm or 3 sccm N_2_ flow. Studies have shown that the strength of implant Ti element may be related to infection and bone resorption [41]. It may be that in different thickness, the trend of element change is different, which affects antibacterial properties.

On the other hand, the antibacterial effect of HfN coating with a thickness of 50 nm against *S. aureus* was better than that of 200 nm, but there was no obvious law in biocompatibility. About 50 and 200 nm films were selected to compare and study the effects of different elemental composition on antimicrobial properties. This is because previous studies have shown that the thickness of the film strongly affects the elemental composition [28]. EDS results show that under the same flux of N_2_, the wt% and at% of Hf increased and that of Ti decreased between 50 and 200 nm thickness. And the HfN coatings with a thickness of 50 nm and 2.5 sccm N_2_ flow rate may be the balance value of the film’s elemental composition and antibacterial properties. It is possible that the Hf wt% is 95.9–96.5, the Hf at% is 66.9–70.3, N wt% is 3–3.5, and the Hf at% is 28.1–31.2, which has the most suitable antibacterial properties and biocompatibility.

However, there were several limitations to our research. First, future studies could further investigate how changes in physicochemical properties – surface roughness, wettability, and surface energy – influence the coating’s biocompatibility and antibacterial efficacy. Second, since we only used one experimental strain as a model, these materials may not be suitable for other species of bacteria, such as methicillin-resistant *S. aureus*. Third, biocompatibility was assessed only at the cellular level, which requires more rigorous animal studies and will be done in the future.

## 5 Conclusions

This is the first study to explore coating the surface of Ti implants with HfN by magnetron sputtering technology. We investigated the antibacterial properties and biocompatibility of the coatings. *In vitro* experiments showed that the HfN coating with a thickness of 50 nm, N_2_ flow rate of 2.5 sccm, and Ar flow rate of 80 sccm had a notable antibacterial effect against *S. aureus* and was not toxic to BMSCs. The surface element of coatings had a significant variation between 50 and 200 nm thickness, and its antibacterial response between 50 and 200 nm thickness also changed accordingly. Further research could explore the effects of surface characteristics (roughness, surface energy, wettability) on the coating’s biocompatibility and antibacterial properties. This study provides a new direction for the design and preparation of antimicrobial coatings for orthopedic implants. However, more experiments and *in vivo* studies are needed in the future to support this hypothesis.

## Supplementary Material

Supplementary Figure
