# Supplementary figures and images for: Preparation of hafnium nitride-coated titanium implants by magnetron sputtering technology and evaluation of their antibacterial properties and biocompatibility
Source: Open Life Sci. 2025 Jul 24;20(1):20251132. doi: 10.1515/biol-2025-1132 (PMC12290375; doi:10.1515/biol-2025-1132)

# Supplementary material

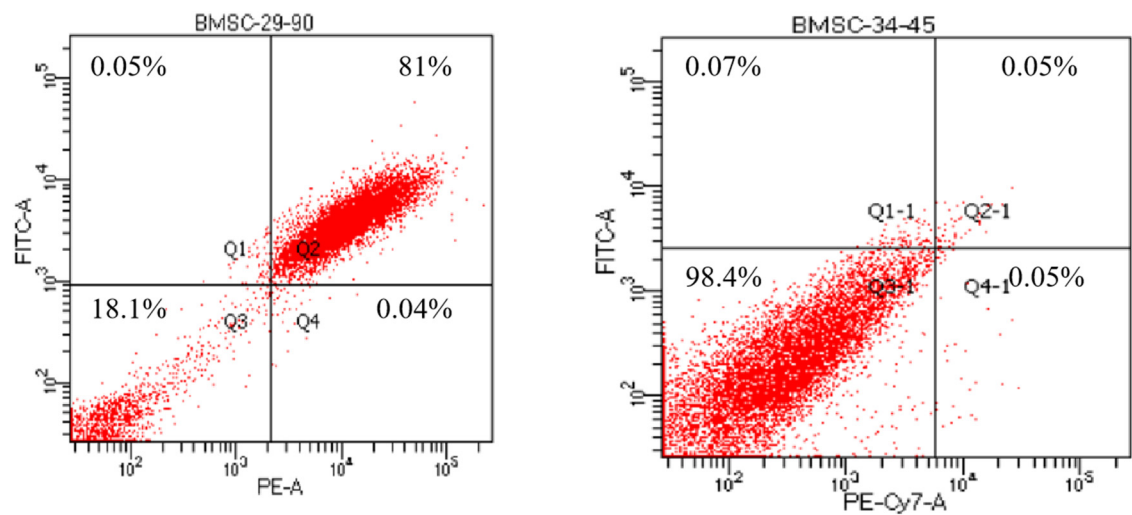

**Figure S1:** Flow cytometry to identify bone mesenchymal stem cells.

Supplement: Supplementary Figure [file biol-2025-1132-sm.pdf]
